# Case report: Two case reports of cryptogenic brain abscess caused by *Fusobacterium nucleatum* and literature review

**DOI:** 10.3389/fnins.2023.1248493

**Published:** 2023-11-23

**Authors:** Zuoxin Zhang, Jing Liu, Linxi Su, Weiwei Huang, Yuchun Pei, Guohao Huang, Lin Yang, Shengqing Lv, Jinbo Yin, Guolong Liu

**Affiliations:** ^1^Department of Neurosurgery, Xinqiao Hospital, Third Military Medical University (Army Medical University), Chongqing, China; ^2^Department of Internal Medicine, Army 953 Hospital, Shigatse Branch of Xinqiao Hospital, Third Military Medical University (Army Medical University), Shigatse, China; ^3^Department of Pathology, Xinqiao Hospital, Third Military Medical University (Army Medical University), Chongqing, China; ^4^Department of Clinical Laboratory, Xinqiao Hospital, Third Military Medical University (Army Medical University), Chongqing, China

**Keywords:** cryptogenic brain abscess, *Fusobacterium nucleatum*, diagnosis, minimally invasive, treatment, limb dysfunction

## Abstract

Brain abscess originates from a localized cerebritis area of brain parenchyma, remaining a refractory infectious disease in the central nervous system. Causative pathogens can be wide-ranging, including bacteria, fungi, or parasites; thus, precise pathogen identification and individualized antimicrobial therapy determine patients’ outcomes. Here, we report two cases where both patients only presented with limb dysfunction, but without symptoms, signs, or biological evidence of infection. Samples were obtained through brain stereoscopic surgeries and microbial identifications were performed to confirm the infection of *Fusobacterium nucleatum*. Further appropriate treatments were given, and the patients recovered well. Patient 1 was a 73-year-old male with a 20-day history of left-sided limbs weakness. A brain MRI showed a space-occupying lesion with a heterogeneously ring-enhancement character in the right frontal lobe. This patient underwent puncture biopsy of the lesion with robot-assisted guidance to confirm a brain abscess. Empirical antibiotic therapy was immediately given until the pathogen was identified as *Fusobacterium nucleatum*; thus, he received specific antibiotic therapy with metronidazole and recovered well after treatment. Patient 2 was a 22-year-old female with heart disease history who complained of right-sided limb weakness for nine days. A brain MRI showed a circular enhanced lesion with a thin capsule wall and surrounding edema in the left frontal lobe. This patient underwent puncture drainage of the lesion with robot-assisted guidance and a brain abscess was confirmed. Empirical antibiotic therapy was given until the pathogen was identified as *Fusobacterium nucleatum* and then she also received metronidazole treatment. Her symptoms recovered and the lesion disappeared after 1 month. Hence, we reviewed the diagnosis and treatment of cryptogenic brain abscess caused by *Fusobacterium nucleatum* and highlight that precise neurosurgical interventions and identification of causative pathogens are crucial for the management of brain abscess.

## Introduction

Brain abscess, which originates from a localized cerebritis area of brain parenchyma, manifests an accumulated and encapsulated collection of pus and a mass-like lesion character (Sah et al., 2020; Chen M. et al., 2022). It always occurs in patients with predisposing factors such as severe immunocompromise, disruption of the brain natural protective barriers, and systemic infectious source (Brouwer et al., 2014a,b). The incidence of brain abscess is approximately 0.3 to 0.9 per 100,000 population; in fact, it is probably higher than actually predicted in immunocompromised patients (Brouwer and van de Beek, 2017). However, brain abscess can originate from cryptogenic sources in immunocompetent patients, and is uncommon (Sáez-Llorens, 2003; Gil et al., 2022). Brain abscess has a high mortality rate, with risks of 21% after 1 year and 16% in the next 2–5 years (Bodilsen et al., 2020), remaining a refractory infectious disease in the central nervous system.

Direct extension from contiguous infectious foci and hematogenous spread of pathogens are the main mechanisms of brain abscess formation (LaPenna and Roos, 2019). Causative pathogens mainly consist of bacteria, fungi, and parasites (Bodilsen et al., 2018). Among them, gram-positive bacteria, specifically *streptococci* (aerobic, anaerobic, and microaerophilic) and *staphylococci* account for a large proportion (Corsini Campioli et al., 2022). Accumulating reports and studies have demonstrated that some causative pathogens were rare and uncommon owing to different predisposing factors; hence, the precise identification of causative pathogens and the optimal selection of appropriate antimicrobial therapy determine patients’ outcomes.

*Fusobacterium nucleatum* belongs to the *Fusobacterium* genus and it is an anaerobic, gram-negative, oral commensal, and periodontal pathogen associated with a variety of human diseases (Han, 2015). *Fusobacterium nucleatum–*induced infections are uncommon but potentially severe. Denes et al. reported, in a retrospective study, that abscesses, bacteremia, and bone infections were the most common types, and that abscesses were found in various organs including the brain (Denes and Barraud, 2016). Odontogenic infection is the common route of *Fusobacterium nucleatum–*caused brain abscess (Burgos-Larraín et al., 2022). *Fusobacterium nucleatum* can initiate the infection and can facilitate the aggregation of other anaerobic bacteria, leading to poly-microbial infections; however, it is much easier to treat with appropriate antibiotics when it is exposed into aerobic conditions.

Generally, neurological imaging assessment, neurosurgical procedures including gross resection and stereotactic biopsy, and optimal antimicrobial therapy are crucial for the management of brain abscess. Individualized diagnosis and therapy contribute to patients’ prognoses. Herein, we report two cases of brain abscess caused by *Fusobacterium nucleatum* and review the clinical presentations, diagnosis, and therapeutic strategy, as well as provide experience in the management of cryptogenic brain abscess caused by *Fusobacterium nucleatum.*

### Case presentation

#### Case 1

**Figure 1:**
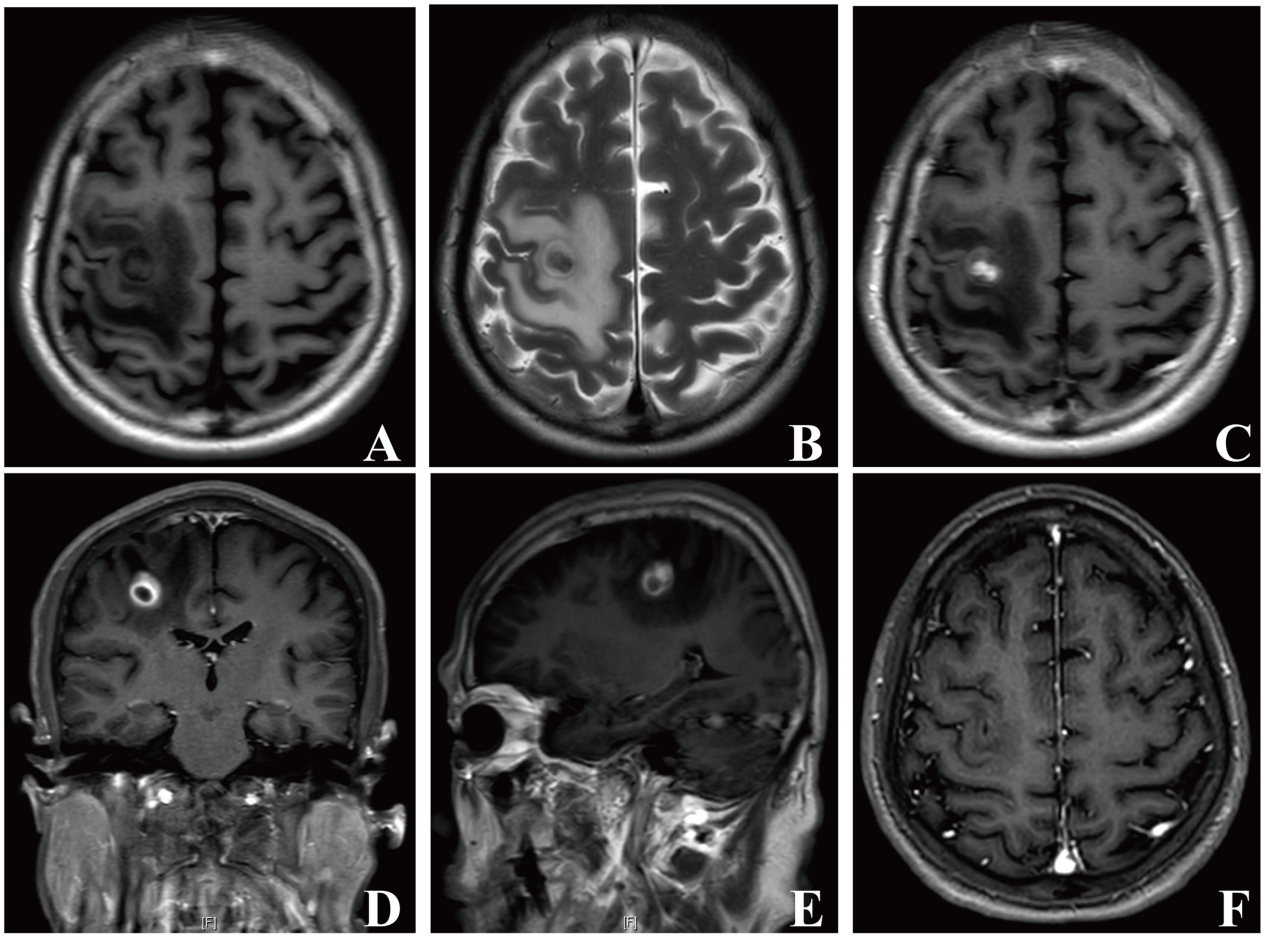
Brain MRI images of patient 1. In the right frontal lobe, a mass-like lesion with peripheral edema presenting long T1 and T2 signals was observed **(A, B)**. Axial, coronal, and sagittal contrast enhanced-T1 weighted images showing a lesion with heterogeneous ring-enhancement **(C–E)**. Repeat brain MRI images showing that the lesion disappeared 1 month later **(F)**.

A 73-year-old male was admitted to our department with a 20-day history of left-sided limb weakness. He complained that his symptoms occurred with no obvious predisposing factors; the severity increased gradually before admission, which remarkably affected his left limb movements. No other neurological symptoms, such as headache, vomiting, vision impairment, or seizure, were present. Physical examination at admission revealed decreased muscle strength of the left-sided limbs; no other abnormal neurological signs were observed. Medical history review identified no record of otitis media, sinusitis, or head trauma and underlying risk factors of brain abscess in elderly people, such as bowel malignancy or pulmonary arterial venous malformation (PAVM), were also not detected at admission. The patient’s body temperature was in the normal range and blood testing showed a leucocyte count of 3.71 × 10^9^/L (normal reference range: 3.5–9.5 × 10^9^/L), 57.8% neutrophils (normal reference range: 40–75%), and 31.8% lymphocytes (normal reference range: 20–50%). Brain magnetic resonance imaging (MRI) showed an aberrant space-occupying lesion with long T1 and T2 signals in the right frontal lobe (Figures 1A,B). Contrast-enhanced imaging manifested a heterogeneous ring-enhancement character with surrounding edema (Figures 1C–E) and no remarkable middle-line shift was present.

**Figure 2:**
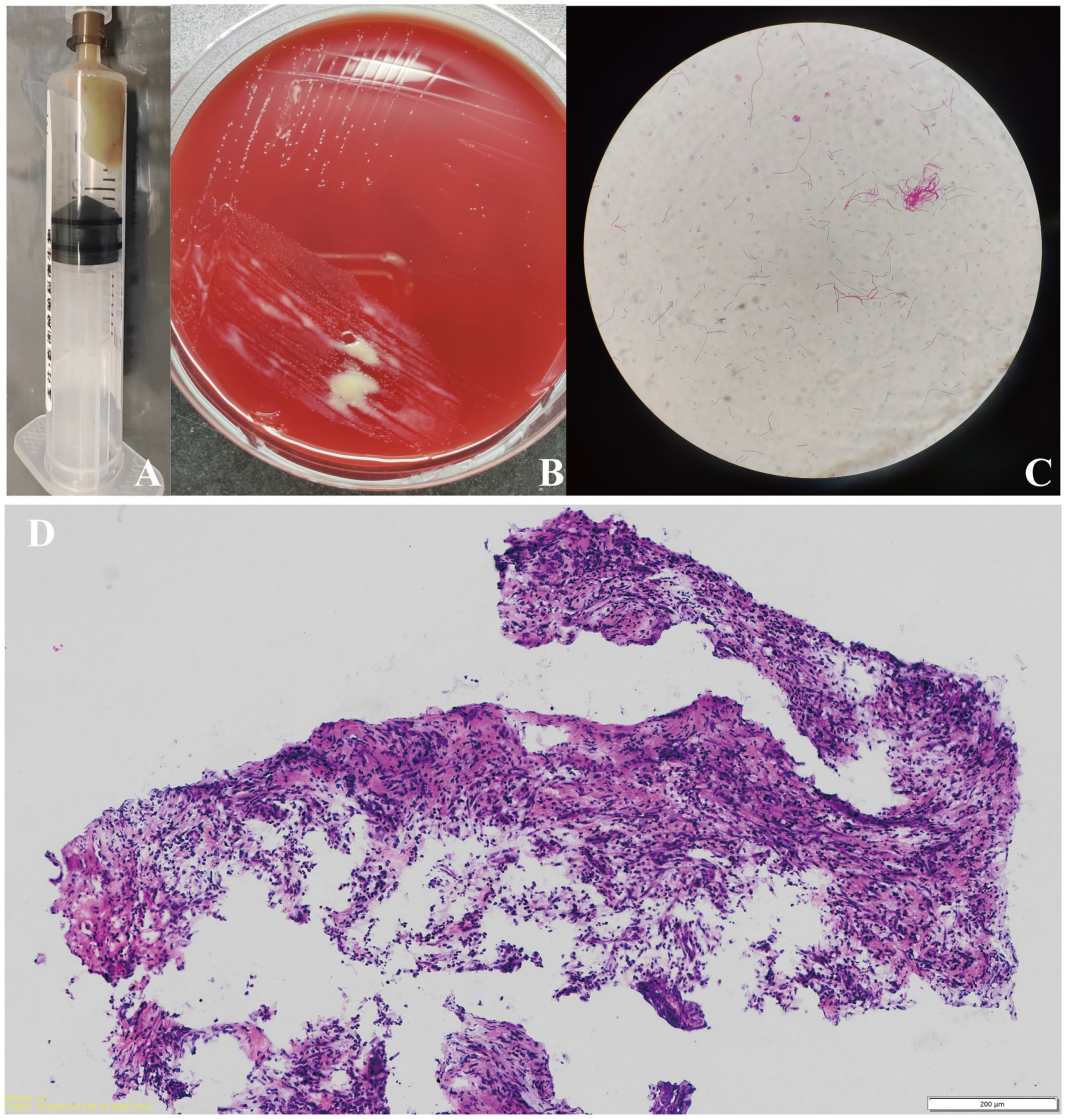
The white-yellow, viscous pus was extracted for pathogen identification **(A)** Images of the bacteria culture of the pus showing that creamy white, moist, and confluent colonies were observed on chocolate agar in an anaerobic medium. **(B)** Gram staining of the extracted bacteria colonies showing gram-negative filamentous rods (10 × 100 magnification). **(C)** Pathological examination of the removed capsule wall tissue in patient 1 showing the presence of inflammation, accompanied with the infiltration of neutrophils (scale bar = 200 μm) **(D)**.

Considering the age and immune status of this patient, a brain glioma was first suspected. A lumbar puncture was then conducted to collect cerebral-spinal fluid (CSF) for testing and microbial culture to exclude brain abscess. The results manifested no bacteria or fungi growth in CSF samples. Moreover, metagenomic next-generation sequencing (mNGS) of pathogens did not detect any microbial DNA fragment. The patient underwent further puncture biopsy of the lesion with robot-assisted guidance and intra-operative frozen pathological examination confirmed an abscess. During operation, a white-yellow, viscous pus was aspirated for microbial culture (Figure 2A) and the capsule wall was removed for pathological examination and mNGS testing. This patient was immediately given empirical antibiotic therapy with meropenem (2 g, q8h, ivgtt) and vancomycin (1 g, q12h, ivgtt) for 7 days until the causative pathogen was confirmed.

The pathological results for the removed tissue indicated the presence of inflammation, accompanied with the infiltration of neutrophils, confirming an abscess (Figure 2D). We thus performed various tests to precisely identify the microorganism. As shown in Figure 2B, creamy white, moist, and confluent colonies were observed on chocolate agar in an anaerobic medium; additionally, the gram staining of extracted bacteria colonies showed the presence of gram-negative filamentous rods (Figure 2C). The microbial culture of pus revealed *Fusobacterium nucleatum* growth. The mNGS results for excised capsule tissues showed a detection of *Fusobacterium nucleatum*. The MALDI Biotyper®, which applies MALDI-TOF MS (matrix-assisted laser desorption/ionization time-of-flight mass spectrometry) technology, identified the isolated bacterial colonies as *Fusobacterium nucleatum* (MALDI score of 2.019). These results confirmed *Fusobacterium nucleatum* to be the pathogenic microorganism of this brain abscess. The patient was prescribed antibiotic therapy with metronidazole (0.5 g, q8h, ivgtt.) and ceftriaxone (2 g, qd, ivgtt.) for 8 weeks. His left-sided limb weakness gradually improved and he had normal muscle strength, body temperature, and clear consciousness when discharged. A follow-up brain MRI showed that the lesion had fully disappeared 1 month later (Figure 1F). A timeline showing the relevant diagnosis and treatment data is shown in Supplementary Figure S1.

#### Case 2

**Figure 3:**
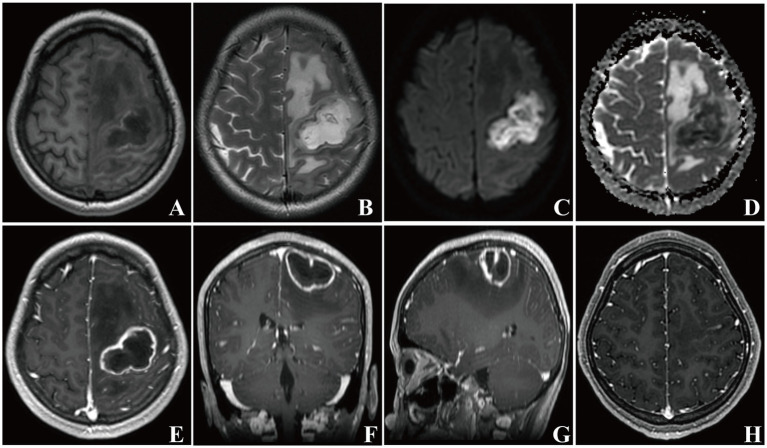
Brain MRI images of patient 2. In the left frontal lobe, a mass-like lesion with peripheral edema presenting long T1 and T2 signals was observed **(A, B)**. A high intensity DWI signal and a low intensity ADC signal of the lesion were present **(C, D)**. Axial, coronal, and sagittal contrast enhanced-T1 weighted images showing a typical ring-enhanced lesion **(E–G)**. Repeat brain MRI images showing that the lesion disappeared 1 month later **(H)**.

**Figure 4:**
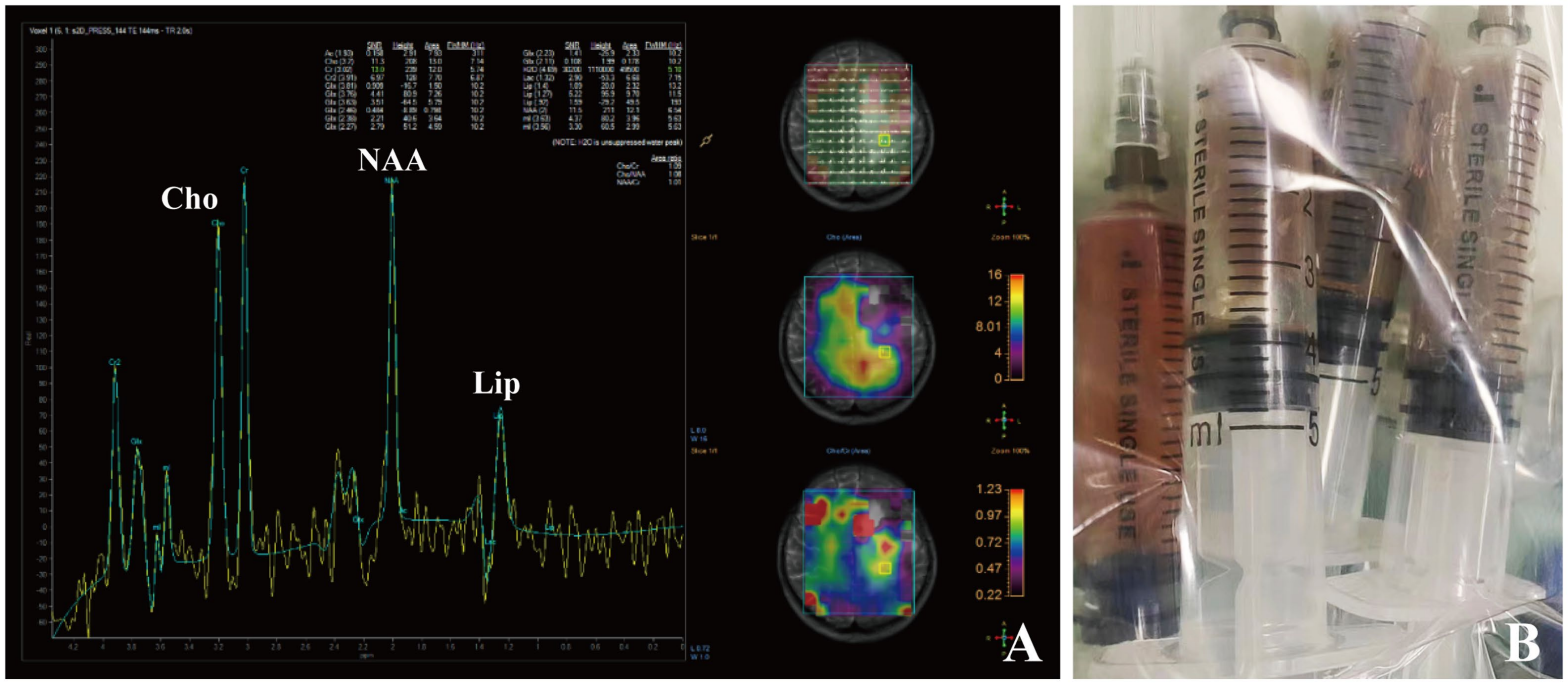
Brain MRS imaging of patient 2 showing an elevated Cho/NAA ratio and amount of lipid in the lesion area **(A)**. Dark-red, viscous pus was aspirated and collected for pathogen identification **(B)**.

A 22-year-old female with a nine-day-history of right-sided limb weakness was hospitalized in our department. Her chief complaint was right-sided limb weakness, accompanied by intermittent limb convulsions. No other abnormal neurological symptoms, such as headache, vomiting, or seizures, were present. Physical examination at admission revealed remarkably decreased muscle strength of the right-sided limbs; no other abnormal neurological signs were observed. This patient had a history of congenital heart disease but she never underwent cardiac surgery; a cardiac ultrasound showed a ventricular septal defect (two-way shunt) and severe pulmonary arterial hypertension, and no clear record of recent infection, sinusitis, or otitis media was identified. The patient’s body temperature was 36.7°C and blood testing showed a leucocyte count of 8.29 × 10^9^/L, 61.9% neutrophils, 30.6% lymphocytes, and C-reactive protein (CRP) levels of less than 5.0 mg/L (normal reference range: 0–8 mg/L). A cranial MRI showed a cystic space-occupying lesion sized 28 × 43 mm with long T1 and T2 signals in the left frontal lobe (Figures 3A,B). High intensely diffusion weighted imaging (DWI) signals and decreased apparent diffusion coefficient (ADC) signals were also manifested (Figures 3C,D). Heterogeneous ring-enhancement and peripheral edema were also revealed in contrast enhanced T1 imaging (Figures 3E–G); additionally, a compressed left ventricle, middle-line shift, and mass effect were observed. Magnetic resonance spectroscopy (MRS) imaging showed an elevated Cho/NAA ratio and amount of lipid in the lesion area compared with the normal region (Figure 4A). Combining these imaging features and her medical history, a brain abscess was highly suspected.

A lumbar puncture was first performed to collect CSF samples for examination. However, both microbial culture and mNGS testing did not indicate the presence of any microorganism in CSF. Considering the location and size of the lesion as well as her heart disease medical history, a neurosurgical procedure was conducted to relieve the compression. This patient underwent puncture drainage of the lesion with robot-assisted guidance. During operation, a copious amount of dark-red, viscous pus was aspirated and collected for microbial culture and mNGS testing (Figure 4B). This patient was immediately given empirical antibiotic therapy with meropenem (2 g, q8h, ivgtt) and vancomycin (1 g, q12h, ivgtt) for 3 days until the causative pathogen was confirmed.

Although the pus culture manifested no bacteria or fungi growth, the mNGS results detected *Fusobacterium nucleatum* DNA fragments in the pus, indicating that *Fusobacterium nucleatum* was the causative pathogen contributing to the brain abscess. This patient was prescribed antibiotic therapy with metronidazole (0.4 g q8h, po.) for 8 weeks. The right-sided limb weakness gradually improved and the convulsions did not occur again. This patient had normal muscle strength and was generally in good condition when discharged; repeat brain MRI imaging showed that the lesion had disappeared after 1 month (Figure 3H). A timeline showing the relevant diagnosis and treatment data is shown in Supplementary Figure S1.

## Discussion

We herein report two typical cases of cryptogenic brain abscess caused by *Fusobacterium nucleatum*. The patients’ sole preoperative clinical presentation was limb dysfunction and no other signs of infection were detected through medical history review, blood testing, or CSF examination. Although brain abscess caused by *Fusobacterium nucleatum* has been reported previously (Lamtri Laarif et al., 2023; Park et al., 2023), this type of cryptogenic brain abscess caused by *Fusobacterium nucleatum* is certainly rare.

Brain abscess remains a refractory, life-threatening infectious disease in the central nervous system. Males account for a 70% of brain abscess cases; the mean age at presentation is approximately 34-years-old (Brouwer and van de Beek, 2017). Its clinical presentations vary, and the most frequent symptoms are headache, fever, and altered level of consciousness (Brouwer et al., 2014a,b). However, cryptogenic brain abscess may often lack these typical clinical manifestations. Focal neurological signs generally depend on the lesion regions; the frontal lobe is reportedly the most common location for an abscess (Corsini Campioli et al., 2021). In our cases, both patients manifested no obvious headache, fever, altered consciousness, or infectious signs, and no clear history of sinusitis, otitis media, or trauma was identified. However, the neurological symptoms, one-sided limb weakness, and decreased muscle strength indicated that the lesion was probably located in the functional area of the frontal lobe. Despite advanced imaging and diagnostic technologies, detailed medical history review and careful physical examination are essential in assisting the diagnosis of brain abscess.

Brain abscesses are often difficult to distinguish from brain tumors radiologically. MRI is valuable in differentiating brain abscess from primary, cystic, or necrotic tumors when combined with DWI and ADC imaging (Brouwer et al., 2014a,b). The typical MRI imaging characteristics of a brain abscess mainly include (i) a high intensity signal with a peripheral rim of low intensity, surrounded by an area of edema with a high intensity signal on T2-weighted imaging, (ii) ring-enhancement of the lesion in contrast-enhanced T1-weighted imaging, (iii) a high intensity signal in diffusion weighted imaging and a low intensity signal in apparent diffusion coefficient imaging at the center of the lesion (De Andres Crespo et al., 2020). Diffusion weighted imaging exhibits high sensitivity and specificity in differentiating brain abscess since an abscess cavity manifests as a hyper-intense signal on DWI with a low ADC value. In contrast, a necrotic, cystic degeneration area of brain tumor displays hypo-intense signals on DWI and high ADC values (Zhou et al., 2019). In our cases, contrast-enhanced T1-weighted imaging of both patients manifested the circular intensification. Patient 1 was given priority for suspicious diagnosis of brain tumor considering his age, and further DWI and ADC imaging were not performed to differentiate brain abscess, which was a shortage in our clinical management. Patient 2 had a medical history of heart disease; thus, we speculated that this patient had a higher probability for the presence of primary infectious foci; typical DWI and ADC imaging did show their ability to differentiate brain abscess from brain tumor. Thus, thorough, multi-modal MRI imaging including DWI and ADC signals is essential for differential diagnosis.

The most common bacteria that contribute to brain abscess are mainly *Streptococcus* and *Staphylococcus* species (Brouwer et al., 2014a,b). The causative pathogens are associated with geographic locations, patient’s age, medical conditions, and primary infection (Su et al., 2023); thus, the spectrum of microorganisms should be considered broad. *Fusobacterium nucleatum* is a gram-negative anaerobic bacteria that exists abundantly in the human oral cavity and gastrointestinal tract (Stokowa-Sołtys et al., 2021; Chen Y. et al., 2022). It often causes opportunistic infections (Brennan and Garrett, 2019) and is associated with various diseases and cancers (Alon-Maimon et al., 2022; Wang and Fang, 2023). This bacterium can enter the blood circulation and can migrate to other parts of the body from periodontal infection (Stokowa-Sołtys et al., 2021). De Andres Crespo et al. summarized the sources of infection that pre-dispose to brain abscess (De Andres Crespo et al., 2020) and reported that *Fusobacterium* species caused cerebral abscesses that were the results of the hematogenous spread of bacteria such as dental infection, bronchiectasis, lung abscess, and empyema. However, the primary infection could not be sourced in some *Fusobacterium–*caused brain abscess cases. Kenig et al. reported a patient with multiple brain abscesses caused by *Fusobacterium nucleatum*; nevertheless, thorough evaluations, including dental examination, a CT scan of the neck soft tissues, transthoracic echocardiography, and a whole-body CT scan, did not reveal a common source for the brain abscess (Kenig et al., 2020). In our cases, although no clear history of infections was manifested and a concrete infectious source in both patients was not identified, we still speculated that the potential source of *Fusobacterium nucleatum* was probably the oral cavity or gastrointestinal tract. Further follow-up examinations and surveillance should be considered to detect the underlying infectious source.

Lumbar puncture plays a limited role in the diagnosis of brain abscess and results from CSF samples may be inconclusive unless the abscess has ruptured into the ventricular system. In our patients, preoperative lumbar punctures were performed, and CSF examinations did not identify any pathogen. The optimal method of identifying the causative microorganism is to acquire the pus of the abscess during drainage for analysis (De Andres Crespo et al., 2020). However, the culture of brain abscess samples is often negative in routine microbiological examination (John et al., 2022). In patient 1, multi-modal methods were utilized to identify the underlying pathogen, including post-operative pus culture, mNGS of pathological tissue, and MALDI-TOF MS of the bacterial colony, which consistently confirmed the infection of *Fusobacterium nucleatum.* In patient 2, the pus microbial culture failed to identify the pathogen, whereas the mNGS detected the infection of *Fusobacterium nucleatum.* We found that the mNGS technology presented high sensitivity and accuracy in identifying the pathogens from the pus sample. mNGS can offer unbiased sequencing results and rapid identification of causative pathogens and is less affected by prior antibiotic exposure (Hu et al., 2019). Hence, analysis of the pus samples not only depends on the routine microbial culture but also on novel molecular methods, which are crucial for the detection and identification of causative pathogens.

The treatment of brain abscess should be comprehensive and neurosurgical procedures and antibiotic therapy are equally essential. The aspiration and drainage of pus can relieve intracranial pressure and allows the acquirement of a pus sample for source identification. Modern, minimally invasive, and stereotactic neurosurgical techniques are helpful for deep or functional region seated brain abscess aspiration and biopsy. In our cases, robot-assisted and image-guided surgery permitted accurate and efficient drainage of the abscesses with minimal risk, and post-operative complications were not present. On the other hand, the early and appropriate choice of an antimicrobial therapeutic strategy determines the outcome of patients and it should be initiated when a brain abscess is suspected since this disease may progress rapidly and unexpectedly. Moreover, pre-operative administration of antimicrobial agents may reduce the yield of bacterial cultures (Brouwer et al., 2014a,b). The initial antimicrobial therapy should be empirical and based on the most likely causative microorganism; additionally, factors such as the infectious mechanism, patient’s predisposing condition, patterns of antimicrobial susceptibility, and the antimicrobial agent’s ability to penetrate the abscess should be taken into consideration (Brouwer et al., 2014a,b). Empiric treatment for immunocompetent patients with brain abscess due to hematogenous spread consists of third-generation cephalosporin combined with metronidazole (Brouwer et al., 2014a,b; De Andres Crespo et al., 2020). For patients who have undergone neurosurgical procedures, vancomycin combined with third– or fourth-generation cephalosporin and metronidazole is usually recommended, and meropenem can be considered as an empirical alternative to cephalosporins or metronidazolein for patients with contraindications (Brouwer et al., 2014a,b). Once the causative pathogen has been isolated, the antimicrobial agents should be modified accordingly to exert their functions more effectively. In our cases, the patients both received empirical antibiotic therapy with meropenem and vancomycin, and pathogen identification revealed *Fusobacterium nucleatum*; thus, the patients were prescribed metronidazole for this anaerobic bacteria. Repeat brain MRI revealed that the brain abscess disappeared and symptoms did not recur after treatment. Nonetheless, long-term follow-up should be performed for such unsourced brain abscess.

## Conclusion

In summary, we have reported two cases of cryptogenic brain abscess caused by *Fusobacterium nucleatum* where the patients only presented with limb dysfunction. Pathogens that contribute to brain abscess have a broad diversity and clinical manifestations are diverse and insidious. Precise neurosurgical interventions and identification of the pathogen, as well as appropriate antimicrobial therapy, determine better outcomes for patients. Preoperative medical history review and laboratory examination of these two patients did not indicate the presence of infection. Through surgical treatment and postoperative microbial identification, cryptogenic infection caused by *Fusobacterium nucleatum* was identified, and the patients recovered well after appropriate antibiotic therapy, avoiding misdiagnosis and mistreatment.

## Data availability statement

The original contributions presented in the study are included in the article/Supplementary materials, further inquiries can be directed to the corresponding authors.

## Ethics statement

The studies involving humans were approved by Ethics Committee of the Xinqiao Hospital of Third Military Medical University. The studies were conducted in accordance with the local legislation and institutional requirements. The participants provided their written informed consent to participate in this study. Written informed consent was obtained from the participant/patient(s) for the publication of this case report.

## Author contributions

GL, YP, GH, LY, and SL cured the patients. LS helped with the pathological diagnosis. WH helped with pathogen identification. GL, JL, JY, and ZZ collected the materials and wrote the manuscript. GL, YP, GH, LS, WH, LY, SL, JL, JY, and ZZ revised the manuscript. All authors contributed to the article and approved the submitted version.
