# Supplementary figures and images for: Case report: Two case reports of cryptogenic brain abscess caused by Fusobacterium nucleatum and literature review
Source: Front Neurosci. 2023 Nov 23;17:1248493. doi: 10.3389/fnins.2023.1248493 (PMC10701533; doi:10.3389/fnins.2023.1248493)

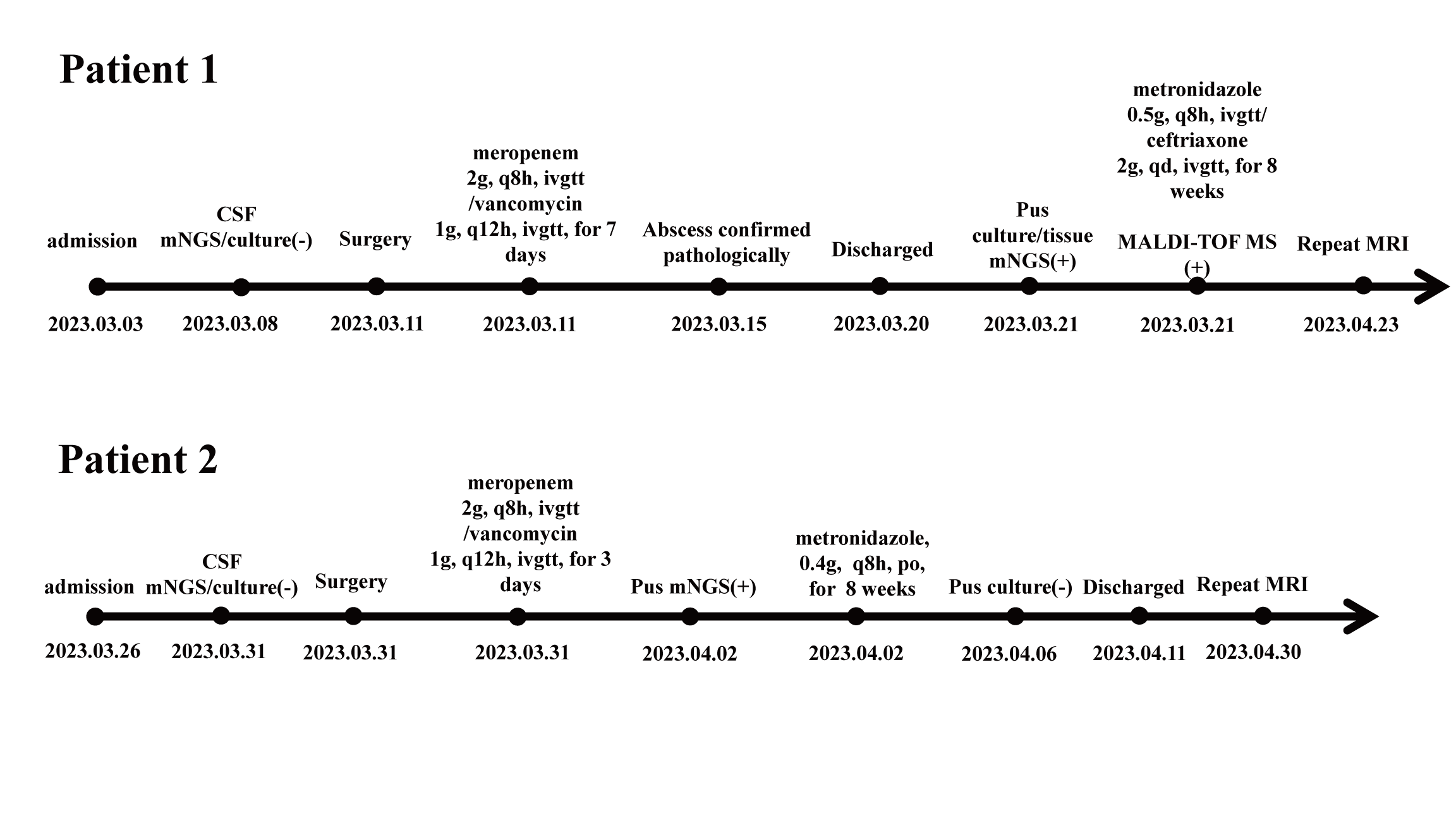

Supplement: Supplementary file 1 [file Image_1.TIF]
